# Shear Bond Strength of Colored and Sandblasted Zirconia to Ceramic Veneers Fabricated by the Pressing and Layering Techniques: An In Vitro Study

**DOI:** 10.1155/2023/4949867

**Published:** 2023-07-18

**Authors:** Mahsa Abbasi, Behnaz Ebadian, Negin Aminianpour

**Affiliations:** ^1^Department of Prosthodontics, School of Dentistry, Shahrekord University of Medical Sciences, Shahrekord, Iran; ^2^Department of Prosthodontics, Dental Implants Research Center, Dental Research Institute, School of Dentistry, Isfahan University of Medical Sciences, Isfahan, Iran; ^3^Dental Research Center, Dentistry Research Institute and Department of Prosthodontics, School of Dentistry, Tehran University of Medical Sciences, Tehran, Iran; ^4^Student Research Committee, Shahrekord University of Medical Sciences, Shahrekord, Iran

## Abstract

**Introduction:**

Porcelain-veneered zirconia (PVZ) restorations are increasingly used due to their optimal esthetics and high strength. However, chipping of porcelain limits the application of PVZ restorations. The aim of this study was to assess the shear bond strength (SBS) of colored and sandblasted zirconia to ceramic veneers fabricated by the pressing and layering techniques.

**Materials and Methods:**

Sixty cubic zirconia specimens (10 × 10 × 2 mm) were assigned to three groups according to their surface treatment: (I) control, (II) sandblasting with 50 *μ*m alumina particles (S), and (III) coloring (C). Each group was subsequently divided into two subgroups according to the porcelain-veneering technique: (I) layering (L) and (II) pressing (P). The specimens underwent 10,000 thermal cycles between 5 and 55°C, and their biaxial SBS was measured in an electromechanical universal test machine (0.5 mm/min with 2.5 kN load cell). The failure mode was also assessed under a stereomicroscope. Three samples were randomly selected from each subgroup (*n* = 18) for examination of zirconia-phase transformation by X-ray diffraction (XRD). Two-way and one-way ANOVA followed by the post hoc Tukey test were used to analyze statistical differences among the groups and subgroups (*α* = 0.05).

**Results:**

The sandblasted zirconia with press porcelain (SP) subgroup showed the highest (24.40 ± 8.16 MPa) and the colored zirconia with press porcelain (CP) subgroup showed the lowest (13.76 ±3.62 MPa) SBS. All failures were cohesive. Rate of phase transformation in layered porcelain was significantly lower than that in pressed porcelain (*P* < 0.01).

**Conclusion:**

The sandblasted group showed the highest and the colored group showed the lowest SBS; the layered group showed higher SBS than the pressed group.

## 1. Introduction

Application of all-ceramic restorations has greatly increased in the recent years due to the increasing attention to esthetics [1]. The primary ceramic restorations did not have sufficient strength especially in the posterior region. However, the advent of computer-aided design and computer-aided manufacturing (CAD/CAM) technology enabled the fabrication of stronger zirconia restorations [2]. Two clinical studies showed no failure and high stability of zirconia-based crowns over a 5-year period [3, 4]. The zirconia core is usually veneered with ceramic because zirconia has low translucency and cannot be easily adjusted due to its high hardness [5]. Porcelain-veneered zirconia (PVZ) restorations provide an esthetically pleasant appearance in addition to sufficient strength. However, porcelain chipping limits the application of PVZ restorations [6].

Several parameters may affect the bond strength of zirconia to porcelain such as porcelain thickness [7], porcelain-veneering technique [8], and type of zirconia surface treatment (such as coloring [9] and sandblasting [10]). However, the results are controversial regarding the effect of sandblasting on bond strength of zirconia. Kim et al. [11] showed that sandblasting significantly increased the shear bond strength (SBS) of zirconia to porcelain; however, another study indicated that the sandblasted group had lower biaxial strength than the control group [12]. Tarib et al. [13] and Ghaffari et al. [14] reported that sandblasting had no significant effect on SBS of zirconia to porcelain. With respect to the effect of coloring of zirconia on its SBS, some studies showed that coloring of zirconia had no significant effect on its SBS [15, 16]; whereas, Ebadian et al. [17] indicated that coloring had a significant adverse effect on biaxial strength.

The results regarding the technique of porcelain veneering on SBS are also conflicting. Ishibe et al. [18] and Kanat-Ertürk et al. [19] showed that the pressing and layering veneering techniques had no significant difference in the control group. In contrast, Juntavee et al. [8] reported that the porcelain layering technique yielded higher SBS.

The crystalline structure of zirconia has three phases of monoclinic, cubic, and tetragonal. Several studies [20–22] concluded that coloring of zirconia had no significant effect on its phase transformation. Inokoshi et al. [23] and Okada et al. [24] stated that sandblasting increased the tetragonal to monoclinic phase transformation. However, Juntavee et al. [8] showed that the layered porcelain veneering group had higher tetragonal to monoclinic phase transformation than the pressed group, but this difference did not reach statistical significance.

Considering all the above, literature is controversial regarding the effects of porcelain-veneering technique, extrinsic pigmentation (coloring) of zirconia, and its sandblasting on SBS, and phase transformation of PVZ restorations. Thus, the purpose of this study was to evaluate the effect of zirconia surface treatments (coloring and sandblasting) and porcelain-veneering techniques (pressing and layering) on SBS of zirconia to porcelain and phase transformation of zirconia. The null hypotheses tested in this study were (I) different surface treatments (sandblasting and coloring) would have no significant effect on SBS of zirconia to porcelain or phase transformation of zirconia, and (II) the veneering technique (pressing or layering) would have no significant effect on SBS and phase transformation of zirconia.

## 2. Materials and Methods

Table 1 presents the manufacturing information of materials used in this in vitro study.

### 2.1. Specimen Preparation

Sixty cubic zirconia specimens (10 × 10 × 2 mm) were fabricated from VITA YZ T blanks. They were designed with a CAD/CAM machine and milled by Imes Icore 450i milling machine (CORiTEC 450i; Imes-icore GmbH, Eiterfeld, Germany) according to Juntavee et al. [8].

### 2.2. Surface Treatments

The milled zirconia specimens were randomly assigned to three groups (*n* = 20): (I) control group without any surface treatment, (II) sandblasting (S) with 50 *μ*m alumina (Al_2_O_3_) particles, and (III) coloring (C) with VITA In-Ceram YZ coloring liquid. Before sintering, group C specimens were immersed in the coloring liquid for 2 min according to the manufacturer's instructions. Next, all specimens were sintered in a furnace (Zyrcomat T; Vita Zahnfabrik, Bad Säckingen, Germany) with this procedure: to reach 900°C in 10 min then 900 to 1530°C in 5 min and finally held in 1530°C for 120 min with standard cooling. After sintering, the S group specimens were sandblasted with 50 *μ*m alumina (Al_2_O_3_) particles with 0.2 MPa pressure for 10 s at 10 mm distance.

### 2.3. Ceramic Veneering

First, the milled zirconia specimens were cleaned with 10% isopropyl alcohol [16] in an ultrasonic bath, and then the specimens in each of the three groups were divided into two subgroups with respect to the porcelain-veneering technique (*n* = 10):

- Conventional layering technique with VITA VM9 by using a silicon index measuring 4 mm × 4 mm × 2 mm. For calibration of layered specimens, the layering process was performed by only one technician for all specimens.
- Pressed technique with VITA PM9: each zirconia specimen was weighed before and after wax-up to determine the required amount of PM9 porcelain. Then, the cubic wax index (designed with STL file output) measuring 4 mm × 4 mm × 2 mm was placed on the zirconia specimens and invested with VITA PM investment material with 10 mm distance from the ring walls. To remove the wax pattern, the invested ring was placed in an oven. After wax burnout, the rings were immediately transferred from the preheating oven to the press furnace. The porcelain was injected into the mold in DP3000 Ivoclar furnace (Ivoclar Vivadent, Schaan, Liechtenstein).

### 2.4. Aging by Thermocycling

All specimens underwent thermocycling (Delta Tpo2; Nemo, Mashhad, Iran) for 10,000 cycles between 5 and 55°C with a dwell time of 20 s and a transfer time of 10 s.

### 2.5. Measuring the SBS

**Figure 1:**
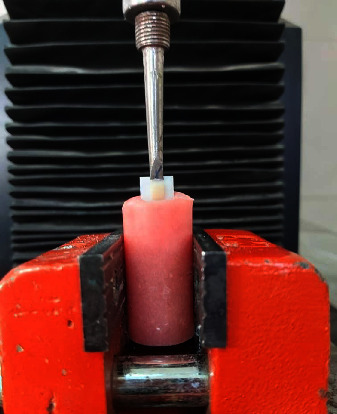
Measuring the SBS of a specimen in an electromechanical universal testing machine.

Fixed specimens underwent the SBS test in an electromechanical universal testing machine (K-21046, Walter + Bai Co, Lohringen, Switzerland) with a 2.5 kN load cell and 0.5 mm/min crosshead speed (Figure 1). SBS calculated through below equation:

(1) $$\begin{array}{c} \text{Shear bond stress }\left(\text{MPa}\right)=\text{load }\left(\text{N}\right)÷\text{area }\left({\text{mm}}^{2}\right) \end{array}$$

### 2.6. Evaluation of Failure Mode

**Figure 2:**
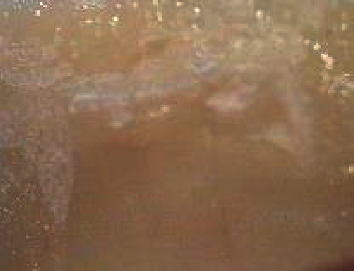
Evaluation of failure mode under a stereomicroscope at ×16 magnification.

The mode of failure was assessed under a stereomicroscope equipped with a digital camera (Trinocular Zoom Stereomicroscope, SMP200, HP, USA) at ×16 magnification (Figure 2), and classified as cohesive in porcelain, cohesive in zirconia, and adhesive between the zirconia and porcelain.

### 2.7. X-Ray Diffraction (XRD) Analysis

**Figure 3:**
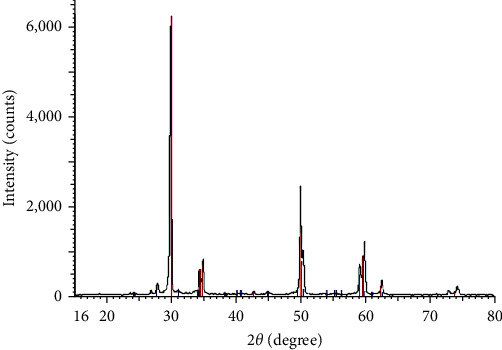
XRD spectra for assessment of the zirconia-phase transformation.

To assess the phase transformation of zirconia, three specimens were randomly selected from each subgroup (*n* = 18). The remaining porcelain was removed with 11% hydrofluoric acid rubbed on the surface by a microbrush. The X-ray settings included 1.54 nm wavelength, 2*θ* = 26–38°, 40 kV, 30 mA, 0.04° step size, and 8 s time for each step (Figure 3). The XRD analysis was performed according to the method described by Garvie and Nicholson [25].

### 2.8. Statistical Analysis

Data were analyzed by SPSS version 20 (SPSS Inc., IL, USA). Two-way ANOVA and Tukey test were used to analyze the effect of surface treatments and porcelain-veneering techniques on SBS and zirconia-phase transformation. One-way ANOVA followed by the Tukey test were used to analyze significant differences in each veneering group. Independent *t*-test was applied to analyze significant differences in each treatment group (*α* = 0.05).

## 3. Results

### 3.1. Evaluation of SBS

**Figure 4:**
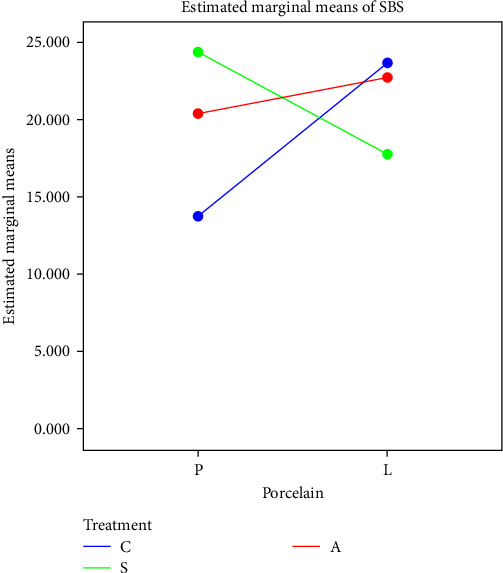
Significant interaction effect of zirconia surface treatment and porcelain-veneering technique on SBS.

According to two-way ANOVA, the interaction effect of porcelain-veneering technique and zirconia treatment on SBS was significant (*P* < 0.01, Figure 4). Therefore, subgroups analysis was performed with independent *t*-test to analyze the effect of veneering technique on SBS in each treatment group, and one-way ANOVA was applied to analyze the effect of surface treatment on SBS in each veneering technique.

Independent *t*-test showed that the difference between CP and CL subgroups, and also SP and SL subgroups was significant (*P* < 0.001 and *P* < 0.05, respectively). One-way ANOVA showed a significant difference among surface treatments in the pressed veneering group (*P* < 0.01).  Table 2 presents the measures of central dispersion for SBS of the study groups.

### 3.2. Evaluation of Failure Mode

Stereomicroscopic examination showed cohesive failure in the veneering ceramic in all specimens in all groups.

### 3.3. XRD Analysis

XRD analysis showed lower percentage of phase transformation in the layering veneering technique (*P* < 0.01) but the effect of zirconia treatment on phase transformation was not significant (*P* > 0.05, Table 3). The CP group showed the highest rate of phase transformation while the CL group showed the lowest rate of phase transformation; this difference was statistically significant (*P* < 0.05, Table 4).

## 4. Discussion

This study evaluated the effect of zirconia surface treatments and porcelain-veneering technique on SBS of PVZ restorations. Sixty zirconia discs were assigned to three groups according to their surface treatment (control, coloring, and sandblasting); next, each group was divided into two subgroups according to the porcelain-veneering technique (pressed and layered). The specimens were evaluated in terms of SBS, mode of failure, and zirconia-phase transformation.

### 4.1. SBS

Sandblasting and coloring of zirconia had no significant effect on SBS of zirconia to layered porcelain (*P* > 0.05). Similarly, Kim et al. [12] and Tarib et al. [13] stated that sandblasting had no significant effect on SBS of zirconia to layered porcelain. However, Kim et al. [11] reported that sandblasting significantly increased the SBS of zirconia to the veneering porcelain applied by the layering technique; this difference may be due to variations in the sandblasting protocol since they used 110 *μ*m alumina particles with 0.4 MPa pressure for 10 s for sandblasting. Also, Ghaffari et al. [14] showed higher SBS in the group sandblasted with 110 *μ*m alumina particles with 3.5 bar pressure for 5 s. Thus, it appears that sandblasting with larger alumina particles and higher pressure may increase the SBS. In a study by Okada et al. [24] the highest SBS value was observed in the sandblasted group with 0.25 MPa pressure. However, sandblasting with 0.4 MPa pressure significantly decreased the SBS. Therefore, it appears that higher sandblasting pressure may increase the SBS only to a certain extent. With respect to the effect of coloring on SBS of layered porcelain to zirconia, Aktas et al. [15] and Bittar et al. [16] showed that coloring of zirconia specimens had no significant effect on SBS of zirconia to layered porcelain (similar to the present findings).

Sandblasting and coloring of zirconia had a significant effect on SBS of zirconia to pressed porcelain (*P* < 0.01) in the present study. Thus, it may be concluded that the pressed veneering technique is more affected by the zirconia surface treatment; however, further studies are required to better elucidate this topic. Ebadian et al. [17] showed that coloring of zirconia specimens significantly decreased the biaxial strength of zirconia to pressed porcelain through slow tempering. Song et al. [26] stated that sandblasting with 110 *μ*m alumina particles and 0.4 MPa pressure and coloring had no significant effect on SBS of zirconia to pressed porcelain in comparison with the control group. This difference may be due to the fact that they did not perform thermocycling in their study. Variations in the brand of products used may also explain the differences in the results.

The pressing and layering–veneering techniques in the control group had no significant effect on SBS. Porcelain-veneering techniques had a significant effect on SBS except in the control group in studies by Ishibe et al. [18] and Kanat-Ertürk et al. [19] who showed no significant difference in SBS between the pressing and layering porcelain-veneering techniques in the control group. However, these findings were different from the results of Juntavee et al. [8] who indicated that layered porcelain had higher SBS; this difference may be due to the use of a liner in their study. Also, the layering technique depends on the experience and skills of the technician. However, in slow tempering groups (similar to the present study), the difference was not significant.

### 4.2. Failure Mode

All failures was cohesive within the porcelain in the present study, which was similar to the results of Ebadian et al. [17] and Roy et al. [27]. This mode of failure may be due to the difference in coefficients of thermal expansion of zirconia and porcelain. Also, zirconia has low-thermal conductivity; thus, it may result in insufficient heat distribution in porcelain firing. In the study by Kim et al. [12] none of the specimens experienced a cohesive failure, which may be due to the application of liner. Also, Shillingburg et al. [28] discussed that application of liner may enhance thermal distribution and result in complete porcelain firing.

### 4.3. XRD Analysis

With respect to zirconia-phase transformation, the present results indicated that the first null hypothesis of the study was accepted, since sandblasting had no significant effect on phase transformation of zirconia. Also Inokoshi et al. [23] and Okada et al. [24] showed higher percentage of zirconia-phase transformation in the sandblasted group, but this difference did not reach statistical significance, probably due to small sample size.

Also, coloring of zirconia specimens had no significant effect on phase transformation of zirconia. Similarly, Tuncel et al. [29] stated that coloring of zirconia resulted in higher percentage of zirconia-phase transformation, but this difference was not significant. Similar results were reported by Shah et al. [20], Ma et al. [22], and Hjerppe et al. [21].

In terms of zirconia-phase transformation, the second null hypothesis was rejected since the layering–veneering technique caused significantly lower percentage of phase transformation. However, Juntavee et al. [8] reported higher percentage of phase transformation in the layering veneering group. This difference may be due to variations in the brands of veneering porcelain that require different temperatures for firing.

The results of SBS and phase transformation percentage were not completely correlated with each other, but it should be noted that a significant difference was observed between the CP and CL groups in both SBS and zirconia-phase transformation. Therefore, it may be concluded that by increasing the percentage of zirconia-phase transformation from tetragonal to monoclinic, the SBS also decreases. However, more studies are needed in this regard.

Considering the limitations of this study, further studies with a larger sample size are required to find possibly significant differences. Also, future studies should perform cyclic loading, and use different particle sizes for sandblasting and different zirconia and ceramic brands. Furthermore, crown-shaped specimens should be used in future studies to better simulate the clinical setting.

## 5. Conclusion

Considering the limitations of this in vitro study, the following results were obtained:

1. Sandblasting and coloring of zirconia had no significant effect on zirconia-phase transformation.
2. SBS in colored zirconia depends on the veneering technique.
3. SBS in pressed veneering technique depends on the type of zirconia surface treatment.
4. The layering veneering technique caused lower percentage of phase transformation.

Further studies are required to investigate the effect of different veneering techniques, specially the pressed technique, on biaxial strength of PVZ restorations, and the effect of different factors on phase transformation of zirconia. Also, the effect of these factors should be assessed in vivo to better understand the zirconia behavior.

## Figures and Tables

**Table 1:** Manufacturing information of materials used in this study.

Material	Manufacturer
Y-TZP blank	VITA Zahnfabrik, Bad Säckingen, Germany
Coloring liquid	VITA-In-Ceram YZ Coloring liquid, medium, Germany
50 *μ*m alumina particles	Siladent, Dr Bohme & Schops GmbH, Germany
VM9 base dentin 2M2	VITA Zahnfabrik, Bad Säckingen, Germany
Silicone	Muller - A-Silicone, Zahnfabric, Germany
VITA PM investment material	VITA Zahnfabrik, Bad Säckingen, Germany
PM9 2M2P-T	VITA Zahnfabrik, Bad Säckingen, Germany
Hydrofluoric acid 11%	Kimia, Iran
Self-cure acrylic repair material	Bayer UK, Newbury, Germany
Wax	Polywax, Baker Hughes (Baker Petrolite - Coatings), Turkey

**Table 2:** Measures of central dispersion for SBS (MPa) of the study groups.

Groups	Mean ± SD	95% confidence interval	
		Lower bound	Upper bound
CP^A+^	13.76 ± 1.89	9.96	17.57
CL^a^	23.65 ± 1.79	20.04	27.26
SP^B+^	24.40 ± 1.79	20.79	28.01
SL^b^	17.79 ± 1.79	14.18	21.40
AP^C+^	20.40 ± 1.79	16.79	24.01
AL^c^	22.74 ± 1.79	19.13	26.35

AP, control pressed; AL, control layered; CP, colored pressed; CL, colored layered; SP, sandblasted pressed; SL, sandblasted layered; SBS, shear bond strength; MPa, megapascals; SD, standard deviation.  ^*∗*^Different superscripted letters (uppercase and lowercase) indicate that the difference between each treatment subgroups (for different veneering technique) is significant as determined by independent *t*-test (*P* < 0.05). Also, + symbol indicates that the difference between each veneering subgroups (for different treatment) is significant as determined by independent *t*-test (*P* < 0.05).

**Table 3:** Results of two-way ANOVA for the effect of porcelain-veneering technique and zirconia treatment on phase transformation (%Xm) of zirconia specimens.

Source	Type III sum of squares	df	Mean square	F	*P* value
Corrected model	0.02^a^	5	0.000	3.810	0.027
Intercept	0.041	1	0.041	318.345	0.000
Treatment	90.954	2	4.291	330	0.725
Porcelain	0.002	1	0.002	16.157	0.002
Treatment × porcelain	0.000	2	0.000	1.117	0.359
Error	0.002	12	0.000		
Total	0.045	18			
Corrected total	2,499.330	17			

^a^R squared = 0.614 (adjusted R squared = 0.453).

**Table 4:** Measures of central dispersion for zirconia-phase transformation (Xm%) in the study groups.

Group	Mean ± SD	95% confidence interval	
		Lower bound	Upper bound
CP^a^	0.066 ± 0.021	0.013	0.120
CL^a^	0.033 ± 0.005	0.019	0.048
SP^b^	0.056 ± 0.010	0.030	0.081
SL^c^	0.040 ± 0.009	0.018	0.063
AP^d^	0.053 ± 0.003	0.043	0.062
AL^e^	0.036 ± 0.008	0.015	0.057

AP, control pressed; AL, control layered; CP, colored pressed; CL, colored layered; SP, sandblasted pressed; SL, sandblasted layered; SBS, shear bond strength; MPa, megapascals; SD, standard deviation.  ^*∗*^Similar superscripted letters indicate statistically significant differences.

## Data Availability

Test data will be sent upon request.
